# Supplementary material for: PlantMetSuite: A User-Friendly Web-Based Tool for Metabolomics Analysis and Visualisation
Source: Plants (Basel). 2023 Aug 6;12(15):2880. doi: 10.3390/plants12152880 (PMC10421497; doi:10.3390/plants12152880)
Supplement: Supplementary file 1 [file plants-12-02880-s001.zip › Figures S1 and S2.pdf]

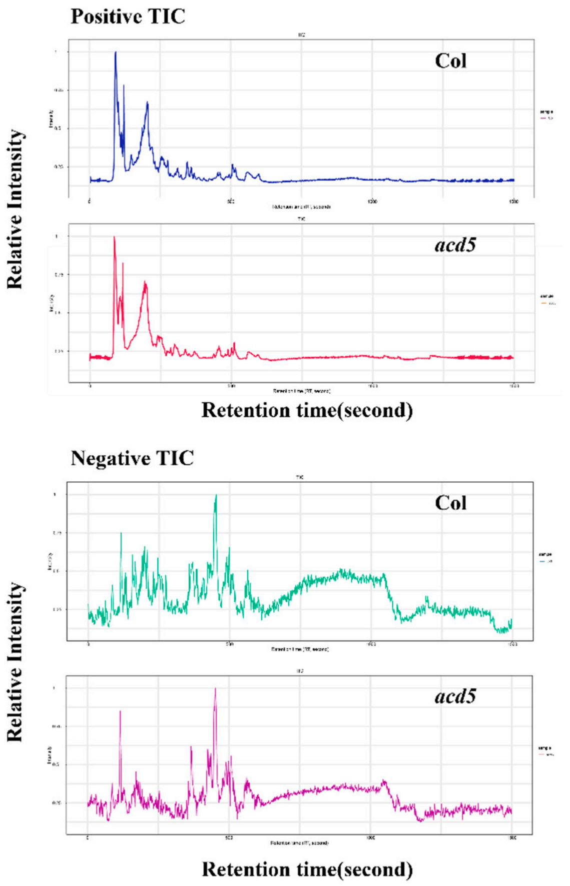

Figure S1. Total ion chromatograms of ceramide kinase mutant *acd5* and wild type Col in positive and negative ion modes. The four coloured lines represent the total ion flow diagram of Col and *acd5* in positive and negative ion modes, respectively.

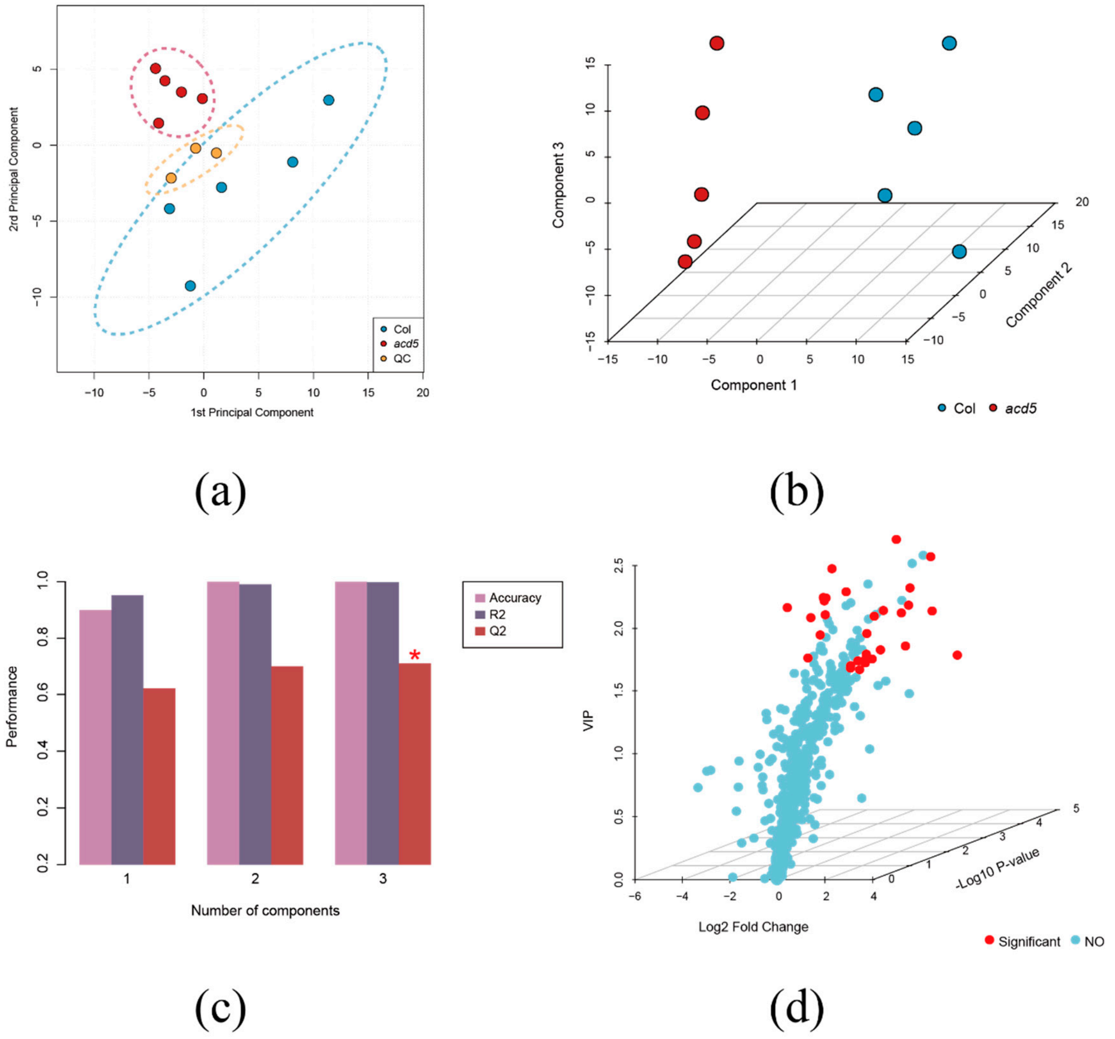

Figure S2. Multivariate statistical analysis of metabolome in *acd5* mutants and wild-type plants. (a) PCA score plot of ceramide kinase mutant acd5 and wild type Col-0. The horizontal coordinate represents the score of PC1, and the vertical coordinate represents the score of PC2. (b) A PLS-DA 3D score plot of acd5 and wild type. (c) PLS-DA classification and feature selection. (d) A three-dimensional scatter plot showing the screened differential metabolites. The red points indicate significantly different metabolites, with the x-axis representing the Log2 fold change, the y-axis representing the -log10 P-value, and the z-axis representing the VIP value
